# Picobirnaviruses encode proteins that are functional bacterial lysins

**DOI:** 10.1073/pnas.2309647120

**Published:** 2023-09-05

**Authors:** Tianyu Gan, David Wang

**Affiliations:** ^a^Department of Molecular Microbiology, School of Medicine, Washington University in St. Louis, St. Louis, MO 63110; ^b^Department of Pathology and Immunology, School of Medicine, Washington University in St. Louis, St. Louis, MO 63110

**Keywords:** picobirnavirus, lysin, RNA phage, bacteria

## Abstract

Picobirnaviruses (PBVs) are double-stranded RNA viruses frequently detected in human and animal enteric viromes. Associations of PBVs with enteric graft-versus-host disease and type I diabetes during pregnancy have been established. Since their discovery in 1988, PBVs have been generally assumed to be animal-infecting viruses despite the lack of culture system, animal model, or detection in animal cells or tissues. Recent studies have proposed that bacteria or fungi could be the hosts of PBVs based on genomic analysis. Here, we functionally demonstrate that multiple PBVs of different genome organizations encode bacterial lysins that lyse *Escherichia coli*. Such genes are typically encoded only by bacteriophages supporting the model that PBVs infect bacterial hosts. Recognition of PBVs as RNA phages in the human gut would completely shift models of how PBVs could impact human health. In addition, expanding the RNA phage world beyond the two recognized clades to three clades has implications for our understanding of the evolution of RNA viruses.

Picobirnaviruses (PBVs) are small, double-stranded RNA viruses belonging to the *Picobirnaviridae* family (1). Human disease associations of PBVs have been established, such as in enteric graft-versus-host disease (2) and type 1 diabetes in pregnant women (3). Since their discovery in 1988 from human stool, PBV sequences have been identified in the fecal samples of many different animals and even in the environment (4). It has been presumed that PBVs are animal-infecting viruses with a wide host range although there is no culture system or animal model. In addition, there has been no detection of viral particles, RNA, or protein within any animal cells.

Recent genomic studies have shown that a high proportion of PBV genomes harbor bacterial ribosomal binding site upstream of their open reading frames (ORFs), which suggested that PBVs could be bacteriophages (5–7). A few PBV genomes have also been identified that use a fungal translational code (7, 8) raising the possibility that PBVs may infect fungi. Given that the underlying mechanisms of diseases would be interpreted dramatically differently depending on whether PBVs infect bacteria, fungi, or animals, it is crucial to clearly understand the biology of PBVs and the nature of their host(s).

A key step toward defining the host would be to identify a PBV protein functionality unique to one of the hosts. One critical step in phage lifecycles is the lysis of bacterial hosts which is necessary for release of progeny. Of the two families of known RNA phages, both encode bacterial lysins (9). Therefore, functional evidence of a bacteriolytic protein from PBVs would strongly suggest that they are bacteriophages.

The current reference PBVs are composed of two RNA segments. PBV segment 1 encodes the viral capsid and 1 or 2 other ORFs (ORF1, ORF2) 5′ to the capsid that have unknown function and no homology with known proteins. PBV segment 2 encodes only the RNA-dependent RNA polymerase (RdRp). In addition to the conventional genome organization, there are two other genome organizations identified more recently. One has an unconventional segmented genome whose segment 2 encodes not only RdRp but also 1 or 2 other ORFs (ORF1, ORF2) that have unknown function; the other organization is a nonsegmented genome that encodes the ORFs of unknown function, capsid, and RDRP on the same segment. If PBVs were to encode lysins, logical candidate genes would be the ORF1 or ORF2.

## Results and Discussion

**Fig. 1.**
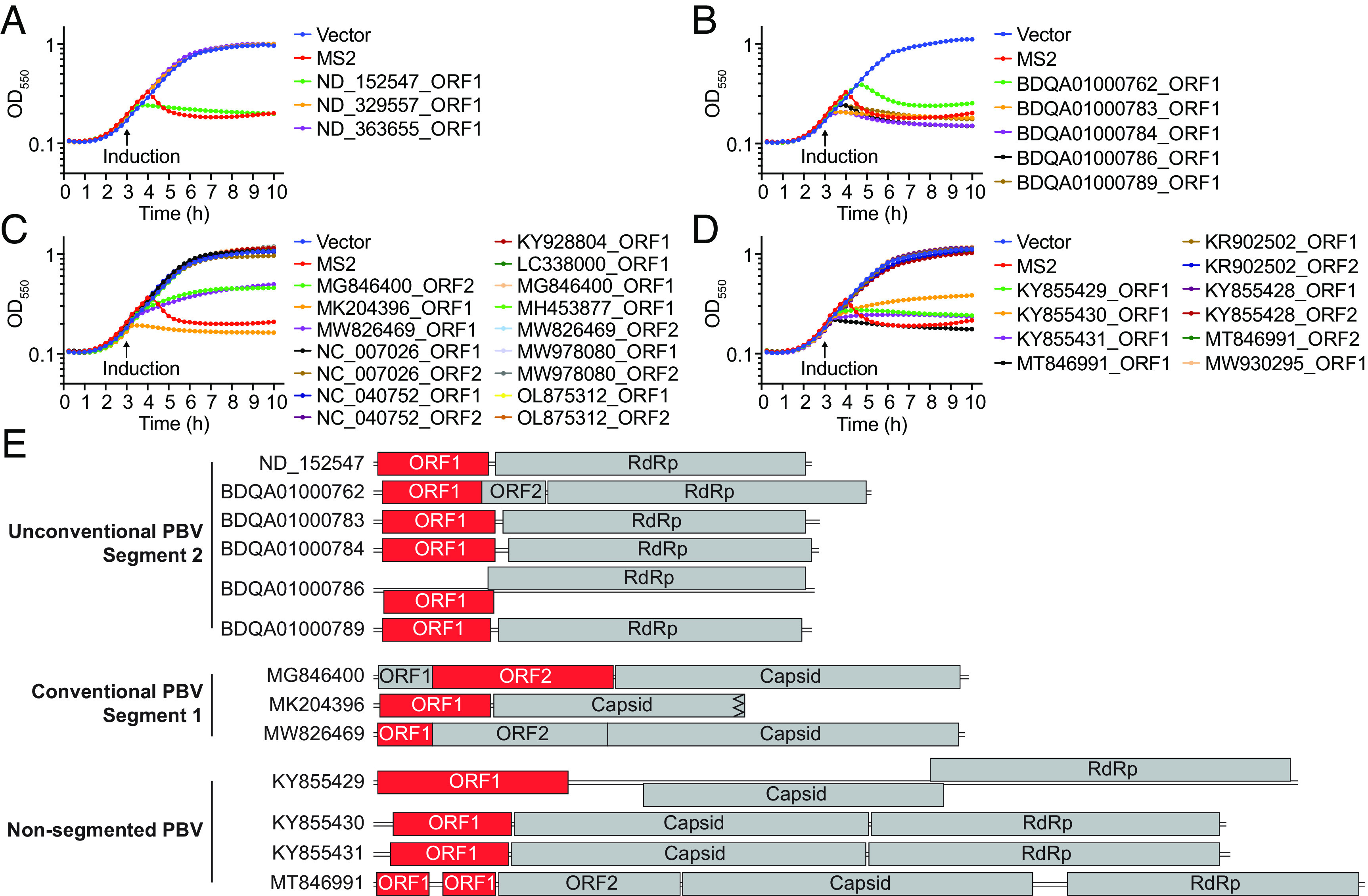
*E. coli* growth profiles with candidate ORFs from PBVs of different genome organizations. (*A*) Growth profiles of *E. coli* with candidate ORFs from unconventional PBV genomes reported in the literature. (*B*–*D*) Growth profiles of *E. coli* with candidate ORFs from unconventional PBV genomes that are selected by having homology with the ORF1 from ND_152547 (*B*), from conventional PBV genomes (*C*), and from nonsegmented PBV genomes (*D*). Empty vector and MS2 lysin were used as the controls. (*E*) The genome structure of PBVs with the functional ORFs shown in red. There are two identical ORF1s in the genome of MT846991. Data are shown in geometric mean of four biological replicates from two independent experiments (*A*–*D*).

Recently, a computational study identified several ORFs from unconventional segmented PBV genomes that have limited similarity to known bacteriolytic proteins, suggesting for the first time that PBVs may encode lysins (10). To determine whether these putative lysins are functional, we cloned the three candidate ORFs from the study, expressed them in *Escherichia coli*, and monitored cell growth for 600 min. Of the three tested ORFs, ORF1 from ND_152547 PBV dramatically reduced bacterial growth comparable to that of the RNA phage MS2 lysin (Fig. 1*A*). The ND_152547_ORF1 shared 46% amino acid identity to M23 family metallopeptidase, a well-known bacteriolytic protein. To determine whether other PBVs encode proteins with homology to ND_152547 ORF1, ND_152547 ORF1 was used as the query to search against all the available PBV sequences in GenBank. We were able to identify five additional ORFs, from five PBV genomes, that shared 42 to 45% amino acid identity with ND_152547 ORF1. These PBV genomes were all reported in a single study of the marine environment (11), and the five ORFs shared low similarity (36 to 69%) to each other. Expression of all five ORFs in *E. coli* significantly reduced bacteria growth comparable to that of MS2 lysin (Fig. 1*B*).

These six functional PBV ORFs were all from PBVs with an unconventional genome organization wherein the ORF is 5′ to the RdRp in segment 2. We next selected 16 ORFs (either ORF1 or ORF1 and ORF2) from 10 representative reference PBVs with the conventional genome organization to determine whether any of these proteins would alter bacteria growth. Three of 16 ORFs, from chicken, rat, and gray teal PBV, reduced bacteria growth with one showing higher activity than the other two (Fig. 1*C*). We also examined 10 ORFs encoded by the few nonsegmented PBVs. Four of 10 ORFs, from marmot and antelope PBV, greatly reduced *E. coli* growth (Fig. 1*D*). The 13 functional PBV ORFs varied in length from 46 aa to 311 aa, and they were located in multiple positions in PBV genomes: 5′ to the capsid, 5′ to the RdRp or 5′ to ORF2 (Fig. 1*E*). Of note, the functional ORFs from conventional and non-segmented PBVs had no detectable sequence similarity to any known protein domain.

**Fig. 2.**
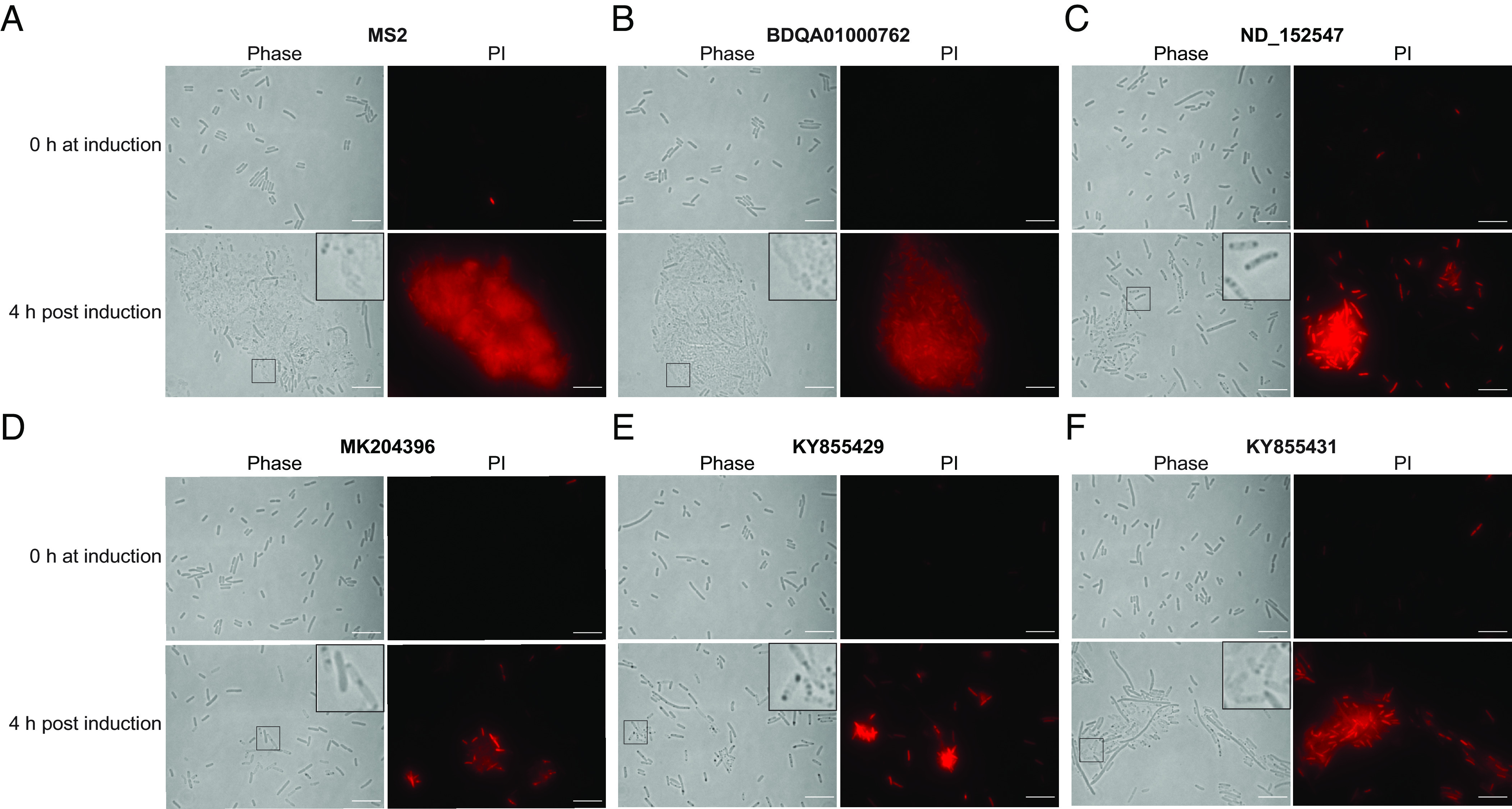
Phase contrast and fluorescence microscopy of bacterial cells before and after induction. Morphologies and PI staining of bacterial cells expressing MS2 lysin (*A*) and representative functional PBV ORFs were shown (*B*–*F*). The zoomed-in images of the lysis morphologies were shown in the insets. Images were representatives from two independent experiments. (Scale bar, 10 µm.)

Next, we used microscopy and propidium iodide (PI) staining to examine the bacterial morphologic phenotypes and membrane integrity resulting from expression of the 13 functional PBV ORFs. PI binds to DNA but is excluded from live, intact bacteria. All the ORFs showed morphological changes by microscopy and loss of membrane integrity by PI staining. There were diverse morphological phenotypes observed, and the representative ones are shown in Fig. 2. First, BDQA01000762 PBV ORF1 caused cell lysis that very closely resembled that of the MS2 lysin (Fig. 2 *A* and *B*). Second, ORFs from ND_152547 (Fig. 2*C*), BDQA01000783, BDQA01000784, BDQA01000786, BDQA01000789, MG846400, MK204396 (Fig. 2*D*), MW826469, KY855429 (Fig. 2*E*), and MT846991 PBV caused lysis and the formation of dark dots in the cell body, which is similar to the phenotype reported for the lysin from phage phiX174 (12). Third, ORFs from KY855430 and KY855431 (Fig. 2*F*) PBV not only caused lysis but also induced extended filaments which mimicked the phenotype that can be caused by peptidoglycan-targeting antibiotics (13). The extensive sequence variation among these PBV lysins, the lack of homology of many of the functional lysins to known domains, and the morphological differences they induce underscore the diversity of bacteriolytic mechanisms in nature (14).

Not all of the tested ORFs were functional in *E. coli*. It is worth noting that phages and their lysins usually have high host specificity (15), so one possibility is that some may function as lysins in other bacterial species. Another possibility we cannot rule out is that some of the PBVs may infect fungi or animals, and the tested ORFs from those genomes may have other functions unrelated to bacterial lysis.

Here, we provide functional evidence that 13 PBVs of different genome organizations all encode bacterial lysins, a key protein function characteristic of bacteriophages, supporting the model that PBVs are RNA phages. There are only two clades of RNA phages known to date, one with positive-sense single-stranded RNA genomes (leviviruses) and the other with trisegmented double-stranded RNA genomes (cystoviruses). The identification of at least a subset of PBVs as RNA phages significantly expands the world of RNA phages. This suggests that RNA phages are far more commonly found in human and animal enteric and respiratory tracts than previously recognized. Moreover, the existence of additional RNA phage taxa is important in the models of the evolutionary history of RNA viruses. For example, one analysis proposed a possible origin for cystoviruses from eukaryotic dsRNA viruses (16); however, the identification of PBVs as RNA phages combined with recent phylogenetic grouping of cystoviruses with PBVs and partitiviruses, enables a more parsimonious prokaryotic origin model for cystoviruses (10). As new experimental and computational evidence accumulates for the existence of additional RNA phage clades, these data will help further refine the models of RNA virus evolution.

## Materials and Methods

XL1-Blue competent cells were transformed with pBAD24 plasmids encoding corresponding ORFs. Growth curves and microscopy were recorded before and after induction of the ORF expression. Detailed descriptions are provided in *SI Appendix*.

## Supplementary Material

Appendix 01 (PDF)

## Data Availability

All study data are included in the article and/or *SI Appendix*.
