# Supplementary material for: Picobirnaviruses encode proteins that are functional bacterial lysins
Source: Proc Natl Acad Sci U S A. 2023 Sep 5;120(37):e2309647120. doi: 10.1073/pnas.2309647120 (PMC10500164; doi:10.1073/pnas.2309647120)
Supplement: Supplementary file 1 — Appendix 01 (PDF) [file pnas.2309647120.sapp.pdf]

Supporting Information

Extended Methods

Plasmids, bacterial strains, and growth conditions

Candidate PBV ORFs were codon-optimized and synthesized by GenScript with restriction site for EcoRI and HindIII. Synthesized ORFs were then cloned with restriction enzymes (New England Biolabs) into inducible vector pBAD24 with DH5α (ThermoFisher Scientific). Plasmid sequences were confirmed by sanger sequencing and transformed into XL1-Blue competent cells (Stratagene) for growth kinetic and microscopy experiments. Cultures were grown at 37 °C with aeration in lysogeny broth (LB) supplemented with ampicillin (100 µg/mL).

Growth kinetics of *E. coli* with candidate PBV ORFs

The *E. coli* growth kinetics were done as described in a previous study (1) with some modifications. Overnight cultures were subcultured at 1:200 (5 µL into 995 µL) in 24-well plates. The plate was incubated at 37 °C with shaking at 548 cpm in Synergy H1 microplate reader (BioTek). OD_{550} was automatically recorded every 15 minutes. The subcultures were induced with L-arabinose (0.4% w/v) when OD_{550} reached 0.2. The incubation and recording were continued in the microplate reader after induction.

Phase contrast and fluorescence microscopy

Overnight cultures were subcultured at 1:200 (12 µL into 2.4 mL) in glass tubes. The tubes were incubated at 37 °C with shaking at 250 rpm. The subcultures were induced with L-arabinose (0.4% w/v) when OD_{550} reached 0.2. Two hundred microliter of bacteria were

pelleted at 0 h at induction and at 4 h post induction and resuspended in 50 µL of PBS containing 1 µg/mL propidium iodide (PI). Low-melting agarose (Lonza) gel pad were made as described in a previous study to immobilize the bacteria (2). Two microliter of the suspension was dropped on top of the gel pad on glass slide and then covered with a coverslip. Phase contrast and fluorescence microscopy were done with the 100× objective of an inverted fluorescence microscope (Leica Microsystems). The same imaging parameters were used for all the samples.

SI References

1. K. R. Chamakura *et al.*, Rapid de novo evolution of lysis genes in single-stranded RNA phages. *Nat Commun* **11**, 6009 (2020).
2. S. O. Skinner, L. A. Sepulveda, H. Xu, I. Golding, Measuring mRNA copy number in individual Escherichia coli cells using single-molecule fluorescent in situ hybridization. *Nat Protoc* **8**, 1100-1113 (2013).
